# Genome-Wide Identification, Plasma Membrane Localization, and Functional Validation of the SUT Gene Family in Yam (*Dioscorea cayennensis* subsp. *rotundata*)

**DOI:** 10.3390/ijms26125756

**Published:** 2025-06-16

**Authors:** Na Li, Yanfang Zhang, Xiuwen Huo, Linan Xing, Mingran Ge, Ningning Suo

**Affiliations:** College of Horticulture and Plant Protection, Inner Mongolia Agricultural University, Hohhot 010018, China; ln455468986sl@163.com (N.L.); zhangyanfang@imau.edu.cn (Y.Z.); xln620719@163.com (L.X.); ge15248178936@126.com (M.G.); suoning200@gmail.com (N.S.)

**Keywords:** *Dioscorea rotundata*, sucrose transporter (SUT), molecular docking simulations, gene expression

## Abstract

Yam (*Dioscorea cayennensis subsp. rotundata*,hereafter referred to as *Dioscorea rotundata*) is a staple tropical tuber crop with notable nutritional and economic value. Its development and yield depend on efficient sucrose allocation from source tissues. Sucrose transporters (SUTs), a conserved family of membrane proteins, mediate sucrose loading, translocation, and unloading. Although well-studied in model plants and cereals, SUTs in yam remain largely uncharacterized. This study aims to identify and characterize the *SUT* gene family in yam and explore their roles in sucrose transport and tuber development. We conducted a genome-wide analysis of yam *SUT* genes, including gene structure, subcellular localization, and phylogeny. Molecular docking was used to predict sucrose-binding residues, and qRT-PCR assessed gene expression across tissues and tuber developmental stages. Eight *SUT* genes were identified and classified based on sequence similarity and domain structure. Docking analysis revealed key residues involved in sucrose binding and possible conformational shifts influencing transport. Expression profiling showed that most *SUT* genes, especially in the tuber apex, were progressively upregulated during development, suggesting roles in sucrose unloading and cell expansion. Additionally, functional validation of *DrSUT1* in Arabidopsis thaliana confirmed its involvement in sucrose transport, supporting its role in yam sucrose partitioning. Yam *SUT* genes, especially those highly expressed in sink tissues, are involved in sucrose partitioning and tuber development. These findings provide structural and functional insights into SUT-mediated sugar transport and lay a foundation for improving sucrose utilization and yield in yam and other tuber crops.

## 1. Introduction

Sugars represent the principal assimilates produced through photosynthesis and are translocated from source to sink tissues via the phloem, where they serve as indispensable substrates supporting plant growth, developmental processes, and responses to biotic and abiotic stresses [1] Among them, sucrose is the predominant mobile form of carbon, functioning not only as a fundamental metabolite but also as a pivotal signaling molecule orchestrating a wide array of physiological and developmental pathways [2,3,4]. The precise regulation of sucrose partitioning is critical for plant productivity and environmental adaptability. This process is primarily mediated by two major classes of sugar transporters: the sucrose transporter (SUT) family and the SWEET (Sugars Will Eventually be Exported Transporter) family [5]. SWEET proteins are responsible for facilitating passive sucrose efflux from mesophyll or phloem parenchyma cells, whereas SUTs actively mediate sucrose uptake into the phloem sieve elements through a proton-coupled symport mechanism, thereby enabling long-distance allocation of photoassimilates to sink tissues such as roots, developing seeds, and fruits [6].

Sucrose transporters (SUTs), also referred to as sucrose/H^+^ symporters, belong to the glycoside-pentoside-hexuronide (GPH):cation symporter family, a subfamily within the major facilitator superfamily (MFS) [7,8]. These transporters are highly conserved across higher plant species and play indispensable roles in the long-distance translocation of sucrose, particularly into sink organs such as fruits, seeds, and tubers, which require elevated sugar concentrations to support proper growth and development [3]. Members of the GPH family exhibit broad substrate specificity, transporting small soluble carbohydrates and amphiphilic solutes, typically in conjunction with monovalent cation symport [4,9,10]. However, SUTs are functionally distinct within this family due to their proton-coupled transport mechanism, which harnesses the electrochemical gradient established by plasma membrane H^+^-ATPases to actively translocate sucrose. Structural studies indicate that SUT proteins comprise 12 transmembrane domains, organized into two topologically distinct halves (TM1–6 and TM7–12), which are essential for substrate recognition and efficient transport. In *Arabidopsis thaliana*, nine *SUT* genes (*SUT1–SUT9*) have been identified, among which *SUT1* was one of the first to be functionally characterized [11]. Phylogenetic analyses have divided the *SUT* gene family into five subfamilies, each displaying unique expression patterns and functional specializations. For example, SUT1-type transporters are exclusively present in dicotyledonous species, whereas SUT3 and SUT5 are monocot-specific [12]. In contrast, the SUT2 and SUT4 subfamilies are found in both monocots and dicots, suggesting conserved evolutionary roles [13]. Functionally, SUT1 members are characterized by high-affinity sucrose uptake and are primarily responsible for phloem loading. SUT2 transporters, despite their sequence similarity, are hypothesized to act as low-affinity sucrose sensors or receptors, potentially participating in regulatory processes rather than bulk transport. SUT4 transporters, on the other hand, function predominantly as low-affinity carriers involved in the retrieval of sucrose from the apoplastic space, thereby fine-tuning the distribution of assimilates within plant tissues [14].

In this study, we refer to the plant species using its accepted botanical nomenclature, *Dioscorea cayennensis* subsp. *rotundata*. Despite its substantial nutritional, medicinal, and economic importance, yam remains an underutilized and under-researched orphan crop. Prior research has largely concentrated on germplasm classification, genetic diversity, and the extraction of bioactive compounds, whereas the molecular mechanisms governing tuber development remain poorly understood. Given the central role of sucrose transport in carbon allocation and biomass accumulation, a comprehensive investigation of the sucrose transporter (*SUT*) gene family is essential. Here, we present a systematic characterization of *SUT* genes in yam, encompassing phylogenetic analysis, gene structure elucidation, expression profiling, and their putative involvement in tuber formation. A novel feature of this study is the integration of molecular docking simulations to gain structural insights into the binding affinity, substrate specificity, and conformational dynamics of SUT proteins in complex with sucrose. Through the identification of key amino acid residues and predicted binding pockets critical for sucrose translocation efficiency, our findings provide mechanistic understanding of carbohydrate partitioning at the molecular level. This study bridges the gap between computational structural biology and functional genomics, offering new perspectives on the regulatory architecture of sucrose transport in yam. Ultimately, our research contributes to the foundational knowledge required for molecular breeding and genetic improvement strategies aimed at enhancing tuber yield and quality in yam and other economically important tuberous crops.

## 2. Results

### 2.1. Identification of Members of the SUT Gene Family

The DrSUT family was identified in the *Dioscorea rotundata* genome through an HMM search. To eliminate incomplete sequences, the candidate genes were further analyzed using the SMART database. A total of eight *DrSUT* genes were identified and named *DrSUT1* through *DrSUT8* (Table 1). Bioinformatics analysis of the *DrSUT* genes revealed that the protein sequences encoded by these genes range from 501 amino acids (DrSUT3) to 616 amino acids (DrSUT1), with an average length of 520.375 amino acids. The molecular weight (MW) ranged from 53.65 kDa (DrSUT4) to 66.14 kDa (DrSUT1). The isoelectric point (pI) values ranged from 6.42 (DrSUT1) to 9.19 (DrSUT4), with an average of 8.56. The instability index ranged from 30.15 to 40.47. None of the DrSUT proteins contain signal peptides. The secondary structure of DrSUT proteins is predominantly random coil (37.33% to 47.24%), followed by α-helix (42.53% to 51.19%) and extended chain (10.23% to 12.18%); none of them contain a β-turn (Table S1). The eight proteins encoded by this type of gene are all localized to the cell membrane, suggesting that most *SUT* genes play a regulatory role in the cell membrane.

**Figure 1:**
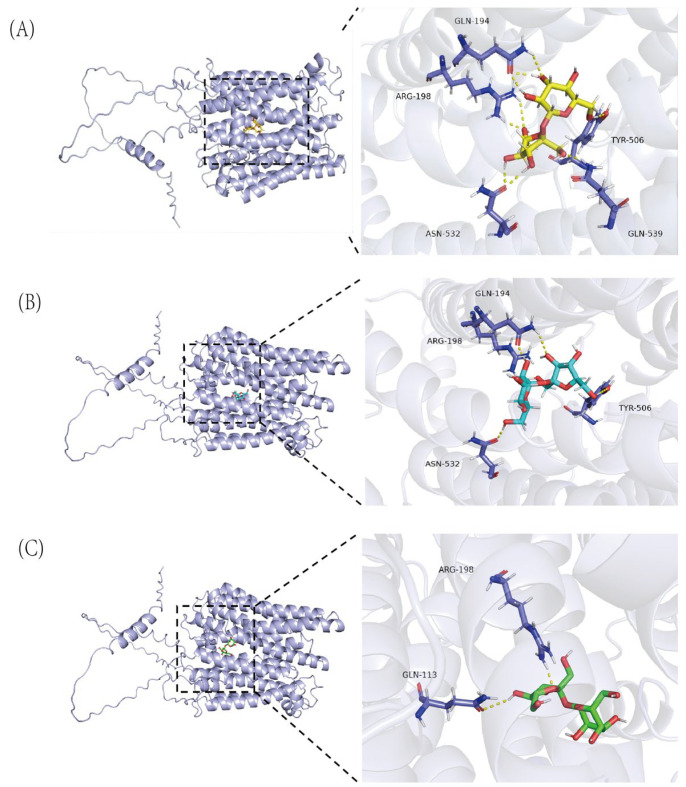
Predicted 3D Binding Models of Sucrose with the SUT Protein via Maestro, DOCK, and AutoDock Vina. (**A**) 3D binding model of the SUT protein (blue) with the compound sucrose (yellow) predicted by Maestro. Key residues are shown as sticks, and hydrogen bonds are represented by yellow dashed lines. Binding energy: −7.868 kcal/mol. (**B**) 3D binding model of the SUT protein (blue) with the compound sucrose (cyan) predicted by DOCK. Key residues are shown as sticks, and hydrogen bonds are represented by yellow dashed lines. Binding energy: −6.158 kcal/mol. (**C**) 3D binding model of the SUT protein (blue) with the compound sucrose (green) predicted by AutoDock Vina. Key residues are shown as sticks, and hydrogen bonds are represented by yellow dashed lines. Binding energy: −6.411 kcal/mol.

We studied the binding modes and interactions between the target protein (SUTs) and the compound (sucrose) through molecular docking. As shown in Figure 1, the protein is represented in cartoon format, and the compound is shown as yellow sticks. The key residues are displayed as sticks. Through docking, we found that the target protein exhibited good binding affinity with the compound (binding energy < −5 kcal/mol). In addition, the compound was able to bind within the active pocket of the protein. Specifically, it formed nine hydrogen bonds with key residues including GLN-194, ARG-198, TYR-506, ASN-532, and GLN-539. These interactions are crucial for maintaining the stability of the protein–ligand complex and may significantly influence their biological function. The binding modes from the three docking software programs are shown in the figure below: yellow (Schrödinger Maestro 2023-2), cyan (DOCK 6.9), and green (AutoDock Vina 1.2.3), with the red region indicating the active site (Figure S1). As shown in the figure, the binding regions of the three modes are closely aligned. Furthermore, all three docking poses are located within the highest-confidence active pocket (Pocket 1). The binding scores obtained from Maestro, DOCK, and AutoDock Vina are summarized in the Supplementary Materials (Table S2). These results further support the consistency of the predicted binding modes and affinities among the three docking tools.

### 2.2. Phylogenetic Analysis and Classification of DrSUT Proteins

**Figure 2:**
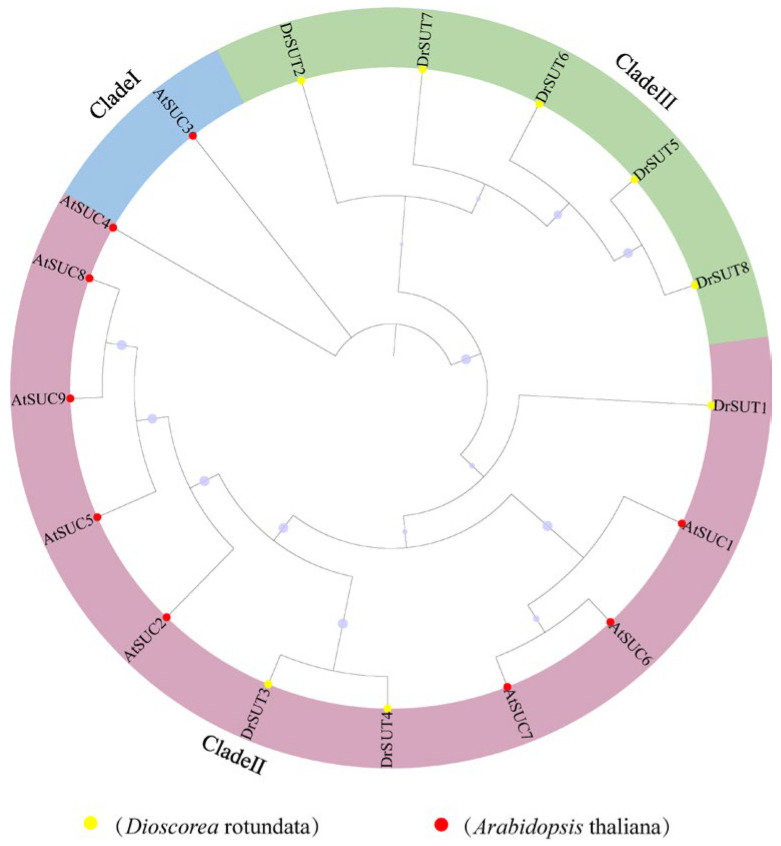
Phylogenetic tree of sucrose transporter (SUT) proteins from *Dioscorea rotundata* and *Arabidopsis thaliana*. The tree was constructed to illustrate evolutionary relationships among the SUT family members. Proteins from *Dioscorea rotundata* are marked with yellow dots, and those from *A. thaliana* are marked with red dots. The tree is divided into three major clades: Clade I (blue), Clade II (green), and Clade III (purple). Colored sectors indicate clade classification. Bootstrap values are shown as purple circles on the branches.

A phylogenetic tree was constructed using 17 SUT proteins, including 8 from *Dioscorea rotundata* (DrSUTs) and 9 from *Arabidopsis thaliana* (AtSUCs), to investigate their evolutionary relationships (Figure 2). Based on sequence similarity, the proteins were classified into three major subfamilies (I–III). Subfamily III contained the largest number of *Dioscorea rotundata* members (DrSUT2, DrSUT5, DrSUT6, DrSUT7, and DrSUT8), followed by subfamily II (DrSUT1, DrSUT3, and DrSUT4). Subfamily I included only AtSUC3 from Arabidopsis, and no *Dioscorea rotundata* SUT proteins clustered into this group. Notably, DrSUTs clustered closely within subfamilies, indicating high structural similarity and a shared evolutionary origin. For example, subgroup II comprised both DrSUT and AtSUC proteins (AtSUC1, AtSUC2, AtSUC4–AtSUC9), suggesting conserved evolutionary trajectories and potential functional similarity between the two species. The phylogenetic distance between subgroups I and III was relatively large, reflecting deeper divergence. Despite this, the overall clustering pattern suggests that DrSUTs and AtSUCs may not have undergone extensive evolutionary divergence and could retain similar biological functions. These results provide a valuable foundation for future functional characterization of the *DrSUT* gene family.

### 2.3. Distribution on Chromosomes, Duplication Events, and Collinear Analysis of DrSUT Proteins

**Figure 3:**
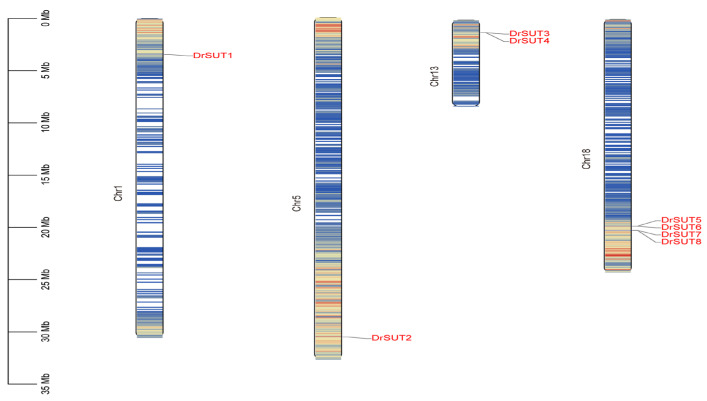
Chromosomal locations and collinearity of *SUT* genes. *SUT* chromosome mapping, where the scale on the left was used to estimate the length of chromosomes and the *SUT* genes of *Dioscorea rotundata* were distributed on four chromosomes.

Chromosome mapping results revealed that the eight *DrSUT* genes are distributed across four chromosomes, with gene numbers ranging from one to four per chromosome (Figure 3). Chromosome 18 harbors the highest number of genes with four (*DrSUT5-8*), accounting for 50% of the total genes, followed by chromosome 13, which contains two genes (*DrSUT3-4*), representing 25%. The chromosomes with the fewest DrSUT genes are chromosomes 1 and 5, each containing only one gene (*DrSUT1* and *DrSUT2*), accounting for 12.5% of the total. This distribution pattern may be related to the gene expansion and functional diversification within subfamilies.

### 2.4. Evolutionary Analysis of DrSUT Proteins and Their Expansion in Several Different Species

**Figure 4:**
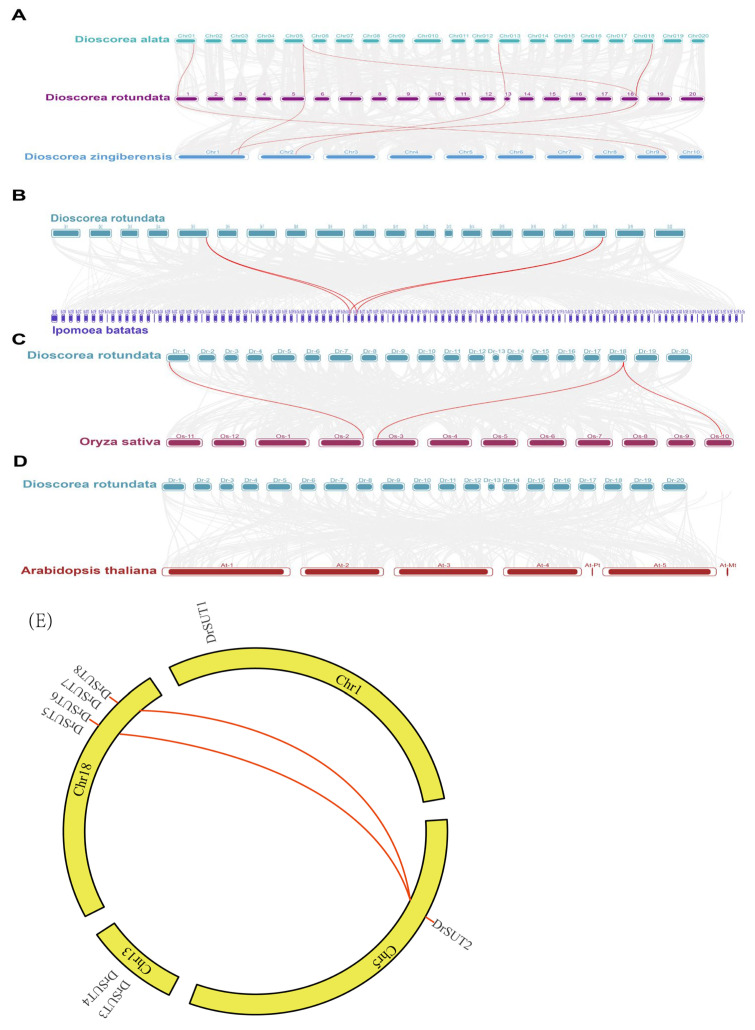
Collinearity analysis map of *Dioscorea rotundata* with *Dioscorea alata*, *Dioscorea zingiberensis* (**A**), sweet potato (**B**), rice (**C**), and *Arabidopsis* (**D**). The red lines indicate collinear gene pairs between the species. The map highlights the genomic similarities between *Dioscorea rotundata* and other species in the *Dioscorea* genus, as well as comparisons with the sweet potato (*Ipomoea batatas*), a member of the Convolvulaceae family, rice (*Oryza sativa*), a monocot, and *Arabidopsis thaliana*, a dicot. Gray lines in the background indicate the collinear blocks within *Dioscorea rotundata* and its progenitor species, while red lines represent the syntenic *SUT* gene pairs. (**E**) Collinearity analysis of *SUT* genes in *Dioscorea rotundata*.

Comparative homology analysis revealed that four *DrSUT* genes exhibit collinearity with *Dioscorea rotundata*, followed by *Dioscorea zingiberensis* and *Dioscorea alata*, with 4 homologous *DrSUT* genes identified in *D. zingiberensis* and 5 in *D. alata*. Additionally, *Dioscorea rotundata* showed four collinear gene pairs with sweet potato (*Ipomoea batatas*) (Figure S2), three with the monocot rice (*Oryza sativa*), and no collinearity with the dicot *Arabidopsis thaliana*. These results suggest a closer genetic relationship with yam species, aligning more closely with monocots than with dicots. This indicates that the synteny between *Dioscorea rotundata* and the yam species reflects a more conserved evolutionary lineage, with greater collinearity observed within the Dioscorea genus and other monocots (Figure 4A–D). Gene duplication is a key factor influencing gene family formation, member expansion, and functional diversification. To better understand the gene duplication events among the *DrSUT* gene family members, we analyzed the duplication events of eight *DrSUT* genes in *Dioscorea rotundata*. A total of two pairs of syntenic genes and three (37.5%) duplicated genes were identified, which are clustered on two chromosomes. Specifically, the duplicated genes are located on chromosome 5 (one cluster) and chromosome 18 (four clusters). These duplicated genes exhibit high homology, and all are segmental duplications. This suggests that the *DrSUT* genes likely originated through gene duplication. Thus, segmental duplication plays a crucial role in the expansion of the *DrSUT* gene family and may be a major driving force in *DrSUT* gene evolution. During the amplification of the *DrSUT* gene family, multiple segmental duplications were observed, but no tandem duplications were detected, indicating a lack of interfering genes at adjacent loci (Figure 4E).

### 2.5. Gene Structure and Motif Composition of the DrSUT Proteins

**Figure 5:**
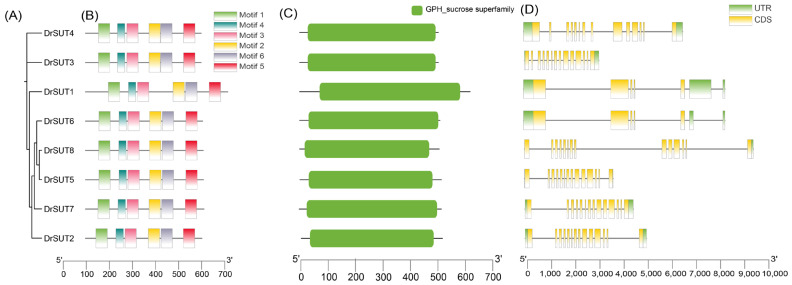
Phylogenetic relationships, conserved motifs, protein domains, and gene structures of *DrSUT* gene family members. (**A**) Phylogenetic tree of the DrSUT protein family constructed based on full-length protein sequences using the method. The DrSUT proteins are grouped into clades within the GPH_sucrose superfamily. Branch lengths represent evolutionary distance. (**B**) Distribution of conserved motifs in DrSUT proteins as identified by MEME. Each colored box represents a distinct conserved motif (Motif 1 to Motif 6). Motif positions are mapped according to amino acid sequence length. (**C**) Conserved domain organization of DrSUT proteins. Domains are represented as color-coded boxes aligned to protein length. Annotations follow the Pfam or InterPro classification. (**D**) Gene structures of *DrSUT* family members, with exons shown as yellow boxes, introns as black lines, and untranslated regions (UTRs) as blue boxes. All genes are displayed from 5′ to 3′ direction and scaled to nucleotide length (see horizontal scale bar).

To investigate the evolutionary and functional characteristics of the *DrSUT* gene family, we analyzed phylogenetic relationships, conserved motifs, protein domains, and gene structures. Phylogenetic clustering (Figure 5A) revealed that members with similar motif compositions tend to group together, suggesting functional similarity within subfamilies. Motif analysis (Figure 5B) identified six conserved motifs mainly located at the N-terminus. While subfamily members generally shared similar motif arrangements, *DrSUT1* exhibited a distinct pattern, indicating possible functional divergence. Domain analysis (Figure 5C) showed that all DrSUT proteins contain a conserved sucrose transporter domain, classifying them within the GPH superfamily. Multiple sequence alignment confirmed the conservation of this domain across the family. Gene structure analysis (Figure 5D) revealed that most *DrSUT* genes have 16 exons and 15 introns, with a few exceptions like *DrSUT3* and *DrSUT4* showing domain loss. Despite variations in exon/intron length, subfamily members displayed highly conserved structures, suggesting structural and functional conservation.

### 2.6. Cis-Regulatory Elements in the Promoters

**Figure 6:**
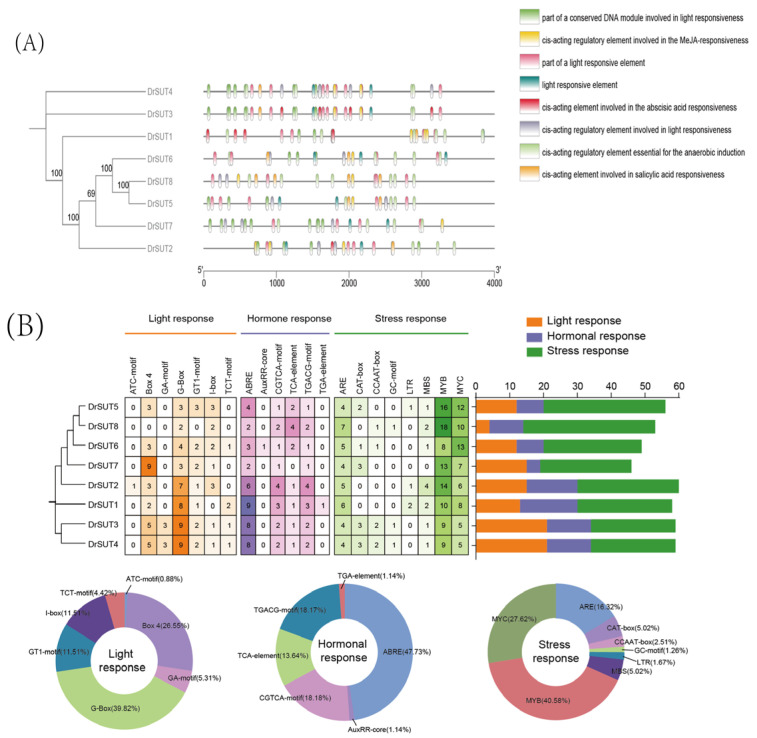
The cis-regulatory elements involved in phytohormone, development, and stress responses in the upstream regions of *DrSUT* gene promoters. (**A**) Analysis of the positional distribution of cis-regulatory elements. (**B**) Statistical analysis of cis-regulatory elements. ARE, involved in anaerobic induction; LTR, low temperature-responsive element; MBS, TC-rich repeats, involved in defense and stress response; G-box, GT1-motif, light-responsive elements; CAT box, GC-motif involved in meristem expression and anoxic specific inducibility, respectively.

We performed a predictive analysis of the cis-regulatory elements in the promoter regions of the *DrSUT* gene family. For *DrSUT1*, the cis-regulatory elements related to abscisic acid (ABA) biosynthesis are most concentrated within the first 1000 bp upstream of the gene (Figure 6A). In contrast, the *DrSUT6* and *DrSUT8* genes exhibit a higher density of cis-regulatory elements associated with light-responsive regulation within the same 1000 bp region. Similarly, the *DrSUT3*, *DrSUT4*, and *DrSUT7* genes harbor elements linked to light-responsive regulation. Light-responsive elements (LREs) are specific DNA sequence modules typically found in plant genomes that can sense light signals and regulate gene expression. Additionally, beyond the 1500–3000 bp region, except for *DrSUT1* and *DrSUT7*, all other genes contain cis-regulatory elements involved in salicylic acid (SA) and jasmonic acid (JA) biosynthesis. With the exception of *DrSUT1* and *DrSUT3*, none of the other genes possess cis-regulatory elements related to ABA biosynthesis. All *SUT* family members harbor cis-regulatory elements involved in light response and those necessary for anaerobic induction. We also predicted and quantitatively assessed the cis-regulatory elements in the 2000 bp upstream region of the *DrSUT* genes (Figure 6B). *DrSUT2* (XM_039269571.1) contains the most cis-regulatory elements (60), while *DrSUT7* (XM_039289167.1) contains the fewest (46). Notably, stress-responsive elements are abundant in the upstream regions of *DrSUT* genes, indicating their regulation by stress response elements. Among light-responsive elements, G-box (39.82%), Box 4 (26.55%), and GT1-motif (11.51%) are the most prevalent, while the hormone-responsive elements include ABRE (47.73%), TGACG-motif (18.17%), and CGTCA (18.18%). Furthermore, stress-responsive elements such as MYB (40.59%), MYC (27.62%), and ARE (16.32%), as well as anaerobic induction elements (ARE, 16.32%; GC-motif, 1.26%), drought response elements (MYB, 40.58%; MYC, 27.62%), low-temperature response elements (LTR, 1.67%), and meristem expression elements (CAT-box, 5.02%) are widely distributed in the promoter regions of *DrSUT* genes. To investigate the functional roles of *DrSUT* genes, we analyzed potential cis-regulatory elements in their promoter regions, focusing on hormone responses, plant growth, and stress responses. The results revealed that most *DrSUT* genes’ promoters contain elements related to hormones such as MeJA (32), ABA (42), SA (12), and IAA (1). We also predicted stress-responsive elements for anaerobic induction (39), drought (12), and low-temperature stress (4), with a significant number of MYB binding sites (97). Additionally, plant growth-related elements like light-responsive elements (113) and meristem expression elements (12) were detected. These findings suggest that *DrSUT* genes are regulated by various cis-regulatory elements during growth, development, and stress responses.

### 2.7. Gene Ontology Annotation Analysis of DrSUTs and DrSWEETs

**Figure 7:**
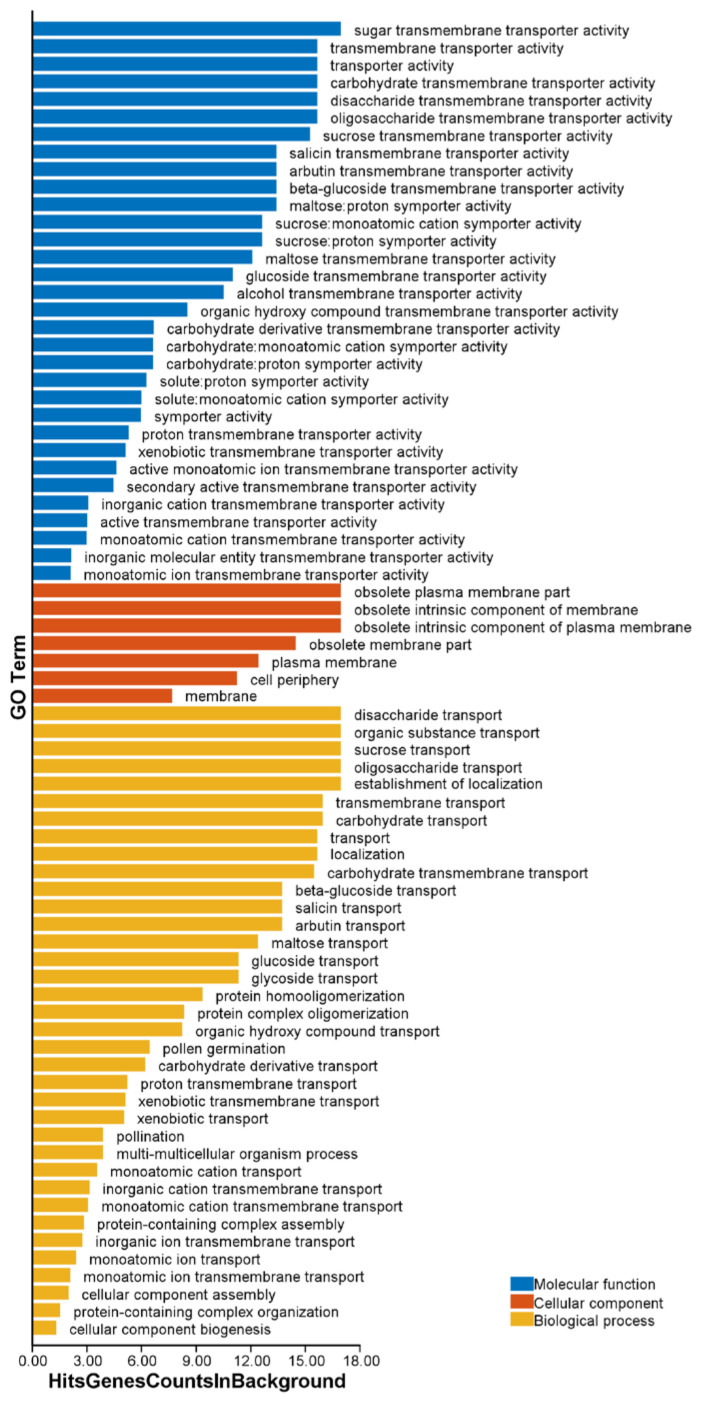
Gene ontology (GO) annotation of the *DrSUTs* and *DrSWEETs*, showing enrichment in the Cellular component, Molecular function, and Biological process categories.

The SUT (Sucrose Transporter) and SWEET (Sugars Will Eventually be Exported Transporters) families play complementary and synergistic roles in the regulation of sucrose transport and distribution within plants. The SUT family is primarily involved in the active transport of sucrose across membranes, facilitating its uptake from the apoplast into plant cells. This function is crucial for sucrose transmembrane transport (GO:0051119), carbohydrate transmembrane transport (GO:0008643), and disaccharide transport (GO:0015774), especially during photosynthesis and sugar translocation from source to sink tissues (Table S3). SUT family members are enriched in terms related to plasma membrane (GO:0005886), emphasizing their role in maintaining membrane integrity. They also act as symporters, such as the sucrose:proton symporter activity (GO:0015702), which couples sucrose transport with proton gradients, enhancing energy efficiency. On the other hand, the SWEET family is responsible for the efflux of sucrose from plant cells to the apoplast, contributing to sucrose transport (GO:0005318) and carbohydrate transport (GO:0015750). Unlike SUTs, which focus on sucrose uptake, SWEETs regulate the release of sugars, balancing the distribution of carbohydrates between source tissues and sink tissues. In addition to sugar transport, SWEET genes play key roles in processes such as pollen germination and plant reproduction (GO:0009853) by controlling sugar availability in reproductive organs. Together, these families collaborate in regulating sucrose distribution, with SUTs facilitating sucrose uptake into cells and SWEETs ensuring its export to surrounding tissues. This coordinated action is vital for plant growth, development, and stress responses. Their synergistic roles are particularly evident in processes like phloem loading and unloading, where the balance of sucrose influx and efflux is critical. Furthermore, their complementary functions in ion balance and proton symporter activity (GO:0015702) enhance cellular processes such as osmoregulation, ion homeostasis, and metabolic stability, reinforcing their mutual dependency in maintaining plant vitality (Figure 7).

### 2.8. Expression Patterns of DrSUT Proteins in Different Tissues and Different Development Stages

**Figure 8:**
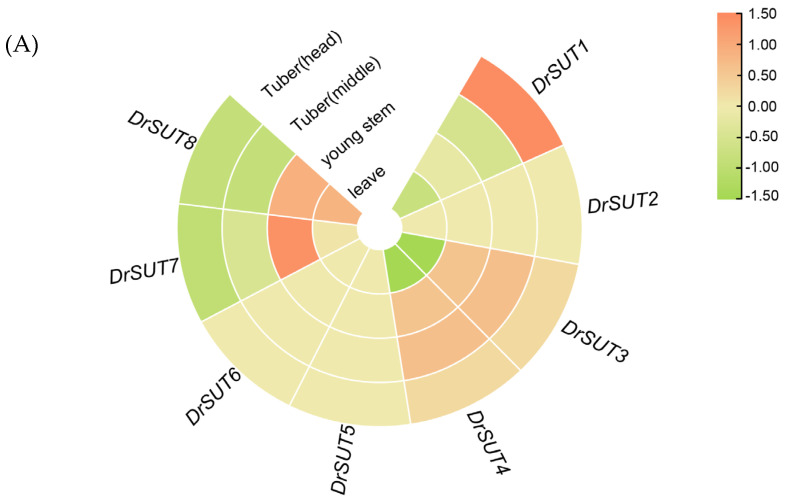

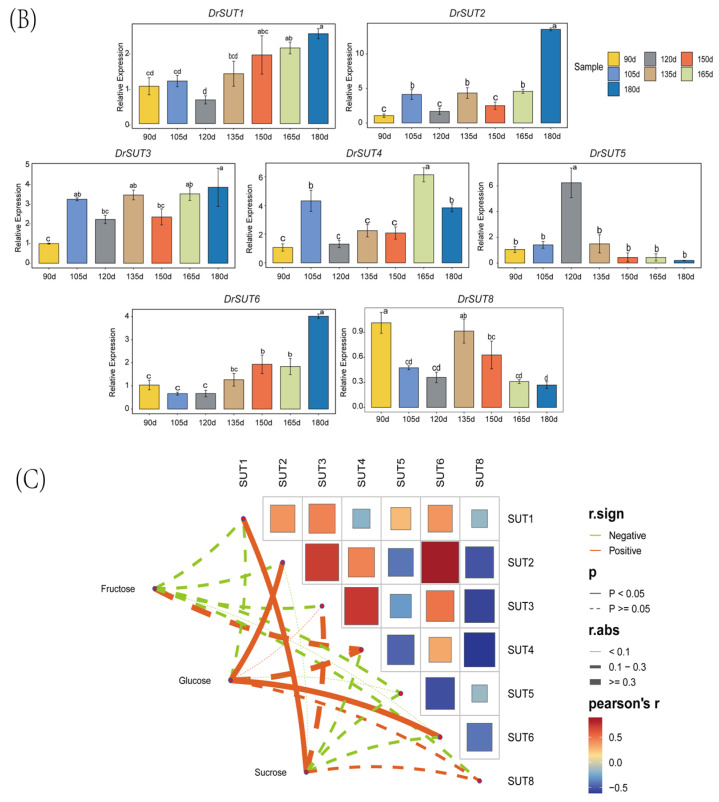
(**A**) Expression profiles of *DrSUT* gene family members in different tissues of *Dioscorea rotundata*. A radial heatmap shows the relative expression levels of *DrSUT1* to *DrSUT8* across five tissue types: tuber (head), tuber (middle), young stem, leaf, and xylem. Color gradients represent log_2_-transformed expression values, with orange indicating high expression and green indicating low expression. (**B**) The relative expression levels of *DrSUT* genes in different developmental phases. Different lowercase letters on the bar indicate significant differences among treatments (*p* < 0.05). (**C**) Bubble-heatmap showing Pearson correlation coefficients (r) between *DrSUT* family gene expression (rows) and sugar contents (columns: sucrose, glucose, fructose) measured in yam tubers at key developmental stages. Bubble color indicates correlation direction (red = positive; blue = negative), bubble size corresponds to absolute correlation magnitude, and bubble border denotes statistical significance (solid border = *p* < 0.05).

Transcriptome analysis revealed distinct tissue-specific expression patterns of the *DrSUT* gene family, indicating functional divergence in sucrose transport. *DrSUT1* was most highly expressed in the apical region of tubers, suggesting a key role in active sucrose transport during early tuber development to support cell division and expansion. *DrSUT2* and *DrSUT3* also showed high expression in tubers, likely contributing to carbon assimilation or sucrose redistribution within storage organs. In contrast, *DrSUT4* and *DrSUT5* exhibited relatively consistent expression across tissues, implying roles in maintaining basal sucrose transport and carbon homeostasis. *DrSUT6* showed very low expression, possibly reflecting limited activity under current conditions or specific functions during stress or particular developmental stages. Notably, *DrSUT7* and *DrSUT8* were significantly upregulated in source tissues such as leaves and young stems, indicating roles in sucrose loading into the phloem and maintaining source–sink carbon flow, as well as potential involvement in energy-intensive processes like cell elongation and hormone signaling (Figure 8A). The expression levels of *DrSUT1*, *DrSUT2*, *DrSUT3*, and *DrSUT6* show a gradual increase at different developmental stages. *DrSUT1* and *DrSUT3* exhibit higher expression levels throughout the entire growth period compared to other stages, indicating that these two transporters play crucial roles. In contrast, the gene expression levels of *DrSUT4* and *DrSUT8* display a declining trend, suggesting their primary function occurs during the early stages of tuber development. Although the expression of *DrSUT5* remains relatively stable throughout the growth period, it peaks at day 120, during the mid-phase of tuber expansion. Among the tuber tissues, all *SUT* genes were expressed, except for *DrSUT7*, whose transcript was barely detectable (Figure 8B). In our correlation analysis between *DrSUT* gene expression and carbohydrate levels across distinct tuber developmental stages, *DrSUT1* expression exhibited a significant positive correlation with sucrose content (Pearson’s r ≈ 0.50, *p* < 0.05), underscoring its primary role in long-distance sucrose transport and local accumulation. *DrSUT3* displayed a moderate positive correlation with glucose levels (r ≈ 0.34, *p* < 0.05), suggesting a potential function in glucose redistribution or metabolic regulation. Similarly, *DrSUT4* was moderately correlated with fructose content (r ≈ 0.30, *p* < 0.05), indicating early signs of functional divergence in fructose transport or sucrose–fructose homeostasis. In contrast, *DrSUT2*, *DrSUT5*, *DrSUT6*, and *DrSUT8* showed weak and largely non-significant correlations with all three carbohydrates (|r| < 0.10 or *p* ≥ 0.05), implying minimal involvement in carbohydrate dynamics at the examined stages. Collectively, these findings not only reinforce the central role of *DrSUT1* in tuber expansion and sucrose accumulation but also provide preliminary evidence for the subfunctionalization of *DrSUT3* and *DrSUT4*, thereby laying a foundation for targeted functional validation of individual family members (Figure 8C).

### 2.9. Subcellular Localization of DrSUT Proteins

**Figure 9:**
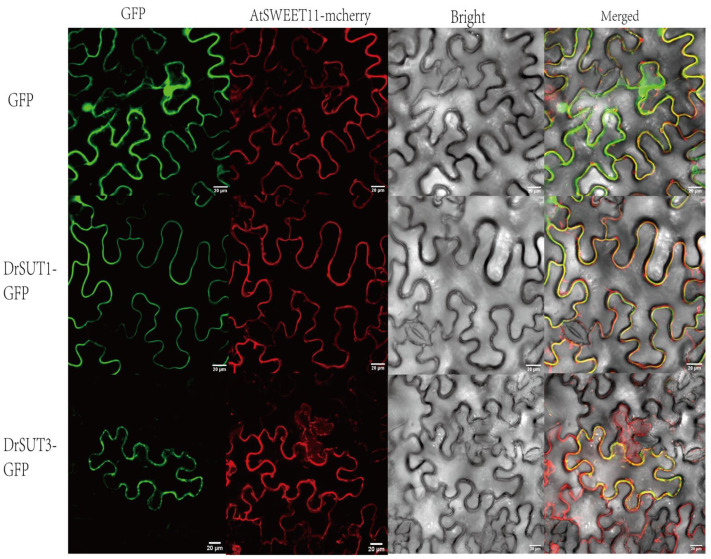
Subcellular localization of two representative SUT proteins. GFP is indicated as empty in the figure, and a membrane localization protein (pCAMBIA1300-35S-PM-mCherry) tagged using co-transformed mCherry was used to visualize the plasma membranes. The fields included the green fluorescence field (488 nm), nucleus autofluorescence field (570 nm), bright field, and merged field. Scale bar is 20 μm.

To determine the subcellular localization of DrSUT proteins, two DrSUT genes (DrSUT1-GFP and DrSUT3-GFP) were cloned into the pCAMBIA1300-GFP vector under the control of the CaMV 35S promoter. The fusion constructs were then transiently expressed in Nicotiana benthamiana epidermal cells and co-expressed with the nuclear localization marker AtSWEET11-mCherry. Fluorescence microscopy revealed that both DrSUT1-GFP and DrSUT3-GFP fusion proteins exhibited specific green fluorescence signals on the plasma membrane (Figure 9). These results confirm that DrSUT1-GFP and DrSUT3-GFP are membrane-localized proteins, consistent with our expectations, and suggest that they may function as transporters.

### 2.10. Functional Validation of DrSUT1 in Transgenic Arabidopsis thaliana

**Figure 10:**
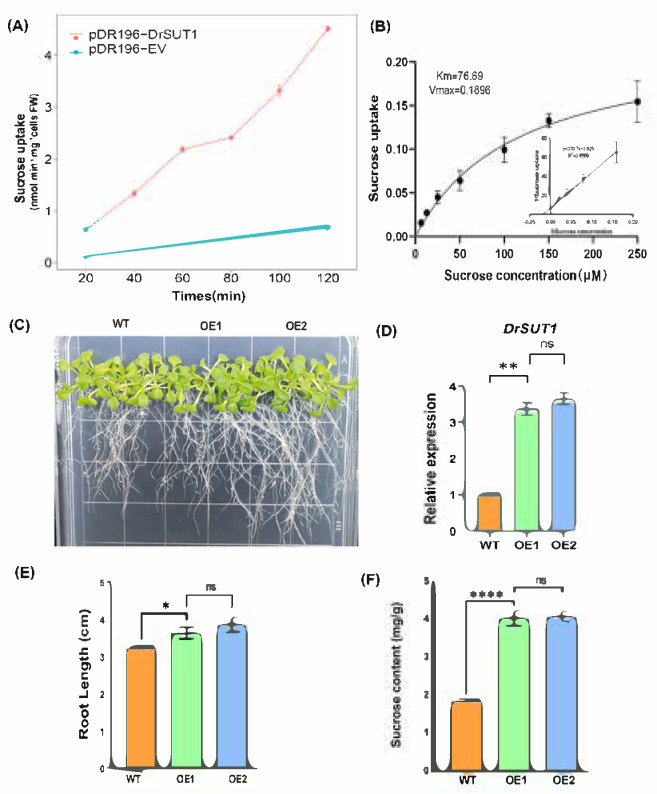
Functional characterization of *DrSUT1* as a sucrose transporter. (**A**) Time-course of sucrose uptake in yeast cells expressing *DrSUT1* (*pDR196-DrSUT1*) versus empty vector (EV). (**B**) Kinetic analysis of *DrSUT*1-mediated sucrose uptake, including nonlinear regression curve and derived parameters (Km and Vmax). (**C**) Lineweaver–Burk plot derived from uptake data in (**B**), confirming linearity and kinetic values. (**D**) Primary root lengths of wild-type (WT), OE1, and OE2 Arabidopsis seedlings grown on MS medium for 7 days. (**E**) qPCR analysis of *DrSUT1* transcript levels in WT, OE1, and OE2. (**F**) Sucrose content in leaves of WT and DrSUT1-overexpressing lines. Data are presented as mean ± SD (n = 3). Asterisks indicate significant differences from WT (* *p* < 0.05, ** *p* < 0.01, **** *p* < 0.0001; one-way ANOVA followed by Tukey’s test).

Yeast cells expressing *DrSUT1* (*pDR196-DrSUT1*) displayed significantly higher sucrose uptake than those carrying the empty vector (*pDR196-EV*), with uptake increasing steadily over time (Figure 10A). In contrast, control cells showed only a slight accumulation. Kinetic analysis revealed a Km of 76.69 μM and Vmax of 0.1896 nmol sucrose·min^−1^·mg^−1^ FW (Figure 10B), supported by a linear Lineweaver–Burk plot (R^2^ = 0.9569). Under normal conditions, *DrSUT1*-overexpressing Arabidopsis lines (OE1 and OE2) exhibited enhanced growth, particularly root elongation. Primary root lengths of WT, OE1, and OE2 averaged 3.21 cm, 3.63 cm, and 4.10 cm, respectively, corresponding to increases of 13.1% and 27.7% in the transgenics (Figure 10C,D). Expression levels of *DrSUT1* were confirmed by qPCR (Figure 10E). Sucrose content in OE1 and OE2 was significantly higher than in WT, averaging 4.04 and 4.09 compared to 1.86 in WT—representing a 2.17- and 2.20-fold increase, respectively. These results indicate that *SUT1* overexpression markedly enhances sucrose accumulation (Figure 10F). One-way ANOVA and pairwise *t*-tests (*p* < 0.001) indicated robust differences between transgenic and wild-type lines, with no significant variation between OE1 and OE2 (*p* = 0.778). Data are shown as mean ± SD (n = 3). Collectively, these results demonstrate that *DrSUT1* encodes a functional sucrose transporter, and that its overexpression enhances sucrose uptake, accumulation, and root growth.

## 3. Discussion

As integral members of the major facilitator superfamily (MFS), sucrose transporters (SUTs) function as proton-coupled symporters that actively mediate sucrose loading into the phloem, unloading into sink tissues, and transport across intracellular membranes such as the tonoplast [15]. Beyond their canonical role in sucrose translocation, SUTs have been implicated in a range of physiological processes, including cell wall biosynthesis, osmotic regulation, and responses to abiotic stresses.

In this study, we identified eight *SUT* family members in *Dioscorea rotundata*. Comparative genomic analysis across diverse plant species revealed notable variation in *SUT* gene family size, with nine members in *Arabidopsis thaliana* [16], five in *Oryza sativa* [17], nine in *Beta vulgaris* [18], and twelve in *Dendrocalamus farinosus* [19]. This interspecific variation likely reflects lineage-specific gene expansion events driven by selective pressures that promote functional diversification in response to distinct developmental or environmental demands [20]. While *SUT* gene function has been extensively characterized in model species and major crops, their molecular roles in tuberous species such as yam remain largely unexplored. To address this gap, we employed molecular docking—a widely used structural biology technique for predicting ligand–protein interactions—to investigate the substrate-binding mechanism of yam SUT proteins [21]. Our docking results revealed that the sucrose molecule forms nine hydrogen bonds with key residues within the binding pocket, including GLN-194, ARG-198, TYR-506, ASN-532, and GLN-539. Specifically, the amide side chains of GLN-194 and GLN-539 contribute to ligand stabilization through hydrogen bonding, while ARG-198 enhances binding affinity via both electrostatic and hydrogen bond interactions. TYR-506 appears to mediate additional stabilization through hydrogen bonding and potential π–π stacking, and ASN-532 reinforces the hydrogen bond network, securing the ligand within the pocket. This extensive network of interactions enhances binding stability, limits ligand conformational freedom, and increases transport specificity [22]. Given the apparent functional importance of these conserved residues, they represent promising targets for genetic manipulation aimed at improving sucrose transport efficiency in yam. Future studies involving site-directed mutagenesis and molecular dynamics simulations could provide further validation of these findings and offer deeper mechanistic insights into SUT-mediated sugar transport.

Phylogenetic analysis grouped *Dioscorea rotundata* SUT (*DrSUT*) genes into three major clades (Groups I, II, and III), consistent with the evolutionary classification of SUTs in *Arabidopsis thaliana* and *Oryza sativa*. Group I members, which cluster with *AtSUT1* and *OsSUT1*, are predicted to function as high-affinity sucrose transporters involved in phloem loading and long-distance assimilate translocation. Group II genes, homologous to *AtSUT2* and *OsSUT2*, are proposed to function as sucrose sensors or regulators rather than primary transporters, as suggested by their lower transport activity and distinctive structural motifs. Group III members, which exhibit greater sequence divergence, are likely associated with intracellular sucrose storage, stress adaptation, or phytohormone-mediated sugar partitioning [23]. Gene duplication has played a central role in the expansion and functional diversification of the *SUT* gene family, enabling neofunctionalization and subfunctionalization across species. Evolutionary analysis revealed that 96% of *SUT* gene pairs in *Cucumis sativus* L. exhibit Ka/Ks ratios < 1 [24], indicating strong purifying selection. A similar pattern was observed in *Helianthus annuus* [25], and this trend appears conserved in *Dioscorea rotundata*, where most *DrSUT* paralog pairs are under strong evolutionary constraint, preserving core sucrose transport functions. Notably, *DrSUT6* displayed a moderately elevated Ka/Ks ratio (Table S3), suggesting potential functional divergence, which may reflect its involvement in enhanced sucrose unloading or redistribution in tuber tissues under specific developmental stages or environmental cues. This hypothesis warrants further validation through transport assays and biochemical characterization. The transcriptional regulation of *DrSUT* genes underpins their tissue-specific and stress-responsive functionality. Promoter analysis identified a rich array of cis-regulatory elements [26], categorized into hormone-responsive motifs (ABRE, AuxRE, GARE, MeJA-responsive elements), implicating *DrSUTs* in hormone-mediated crosstalk during sugar partitioning. Additionally, stress-responsive elements such as LTR (low-temperature responsiveness), ARE (anaerobic induction), and MBS (MYB-binding site for drought inducibility) suggest that *DrSUTs* may contribute to drought tolerance, oxidative stress responses, and sugar starvation signaling. Light-responsive elements were also prevalent, implying regulatory linkage between photosynthetic activity and sucrose transport. Furthermore, the presence of MYB and MYC binding sites in *DrSUT* promoters [27] supports the hypothesis that these genes are transcriptionally modulated by sugar- and stress-responsive transcription factors, in line with mechanisms previously reported for *OsSUT2* in rice and *SlSUT4* in tomato. Recent findings in *Dendrocalamus farinosus* further support this regulatory framework, showing that ABA-responsive elements (ABRE) and MYB motifs contribute to an ABA-dependent feedback mechanism regulating sucrose transporter expression under stress conditions [28]. These regulatory features, conserved across monocots and dicots, underscore the evolutionary importance of transcriptional plasticity in fine-tuning sucrose transport efficiency during plant development and environmental adaptation.

In this study, Gene Ontology (GO) enrichment analysis was employed to systematically investigate the functional roles of the *Dioscorea rotundata* sucrose transporter (*DrSUT*) gene family and to assess their potential synergism with the SWEET transporter family. While the SUT proteins primarily mediate proton-coupled sucrose uptake and intracellular translocation, members of the SWEET family facilitate sucrose efflux into the apoplast, thereby enabling intercellular sugar distribution and maintaining sugar homeostasis [29]. GO enrichment analysis revealed that both transporter families are significantly enriched in terms related to sugar transmembrane transport, carbohydrate translocation, and membrane localization, underscoring their pivotal roles in regulating sucrose flow and maintaining the plant’s energy equilibrium. The complementary functions of SUTs and SWEETs, particularly during phloem loading and unloading, highlight their coordinated activity in orchestrating efficient long-distance sucrose transport [30].

Transcriptome analysis revealed that *DrSUT1* is highly expressed in tuber tissues, indicating its pivotal role in sucrose unloading and accumulation [7]. Further investigations showed that *DrSUT3* and *DrSUT4* also exhibit elevated expression in tubers, suggesting their involvement in long-distance sucrose transport through the phloem, thereby supporting tuber development [31]. In contrast, *DrSUT8* is predominantly expressed in leaves and young stems, implying a function in facilitating source-to-sink sucrose transport [32]. Interestingly, the low expression of *DrSUT2* in tubers suggests a potential role in sucrose signaling rather than transport, supporting the hypothesis that clade II *SUTs* may act as sucrose sensors [33]. Quantitative real-time PCR analysis across different developmental stages revealed that *DrSUT1*, *DrSUT3*, and *DrSUT8* are highly expressed during early tuber development, implicating their roles in initial sucrose partitioning [34]. In contrast, *DrSUT2*, *DrSUT5*, and *DrSUT6* were upregulated at later stages, suggesting functions in sucrose unloading and reutilization during tuber maturation [35]. Subcellular localization studies confirmed that *DrSUT1* and *DrSUT3* are localized to the plasma membrane, consistent with their proposed roles in sucrose import and intercellular transport. To further validate the function of *DrSUT1*, transgenic expression in *Arabidopsis thaliana* demonstrated enhanced sucrose uptake and long-distance translocation, confirming its role as a high-affinity sucrose transporter [36]. These findings not only support the conserved function of *DrSUT1* but also suggest its potential utility in crop improvement strategies aimed at enhancing sink strength and optimizing carbohydrate allocation.

## 4. Materials and Methods

### 4.1. Plant Materials

The experiment involved the cultivation of yam on sandy loam soil at the Inner Mongolia Agricultural University experimental site in the spring of 2023. The field was laid out in uniform planting rows with 80–90 plants per row and 80 cm between rows. Samples, including tubers (head), tubers (middle) stems, and leaves from yam varieties, were collected at various developmental stages (90, 105, 120, 135, 150, 165, and 180 days). Three biological replicates were selected for RNA extraction. The samples, harvested 90 days post-planting, were immediately frozen in liquid nitrogen and stored at −80 °C for further analysis.

### 4.2. Identification of the SUT Genes of Yams

We downloaded the whole-genome and proteome data of “*Dioscorea zingiberensis*”, “*Dioscorea alata*” and “*Dioscorea rotundata*” from the Yam omics (https://biotec.njau.edu.cn/yamdb/, accessed on 18 December 2024) [37]. The SUT protein sequences of *Dioscorea rotundat*a were obtained from NCBI (https://www.ncbi.nlm.nih.gov/datasets/genome/GCF_009730915.1/, accessed on 18 December 2024), and the AtSUTs protein sequences were obtained from the Arabidopsis Information Resource (TAIR, version 10, http://www.arabidopsis.org, accessed on 18 December 2024) [38]. A local protein database was created, and BLASTP (E-value < 1 × 10^−5^) was used to identify potential SUT family members by aligning the sequences. In addition, the HMM file for the SUT domain (PF13347) was retrieved from the Pfam database (http://pfam-legacy.xfam.org/, accessed on 18 December 2024) [39], and HMMER v3.3.2 software was applied to detect possible SUT proteins accessed on 18 December 2024) [40]. Candidate sequences were further verified by submitting them to the SMART (http://smart.embl-heidelberg.de/, accessed on 18 December 2024) [41], and to ensure the integrity of the SUT domain, we compared the identified SUT family members using tools from the NCBI, CDD (https://www.ncbi.nlm.nih.gov/cdd, accessed on 18 December 2024) [42]. To predict the molecular characteristics of the SUT proteins, including length, molecular weight, theoretical isoelectric point, instability coefficient, hydrophobicity, and average hydrophilicity, we used the ProtParam tool (https://web.expasy.org/protparam/, accessed on 18 December 2024) [43]. Additionally, the transmembrane structure, subcellular localization, and secondary structure of the SUT family members in *Dioscorea rotundata* were predicted using TMHMM2.0 [44] (https://services.healthtech.dtu.dk/services/TMHMM-2.0/, accessed on 18 December 2024) Cell-PLoc (http://www.csbio.sjtu.edu.cn/bioinf/Cell-PLoc-2/, accessed on 18 December 2024) and SOPMA (https://npsa-prabi.ibcp.fr/cgi-bin/npsa_automat.pl?page=npsa_sopma.html, accessed on 18 December 2024). Structure of active ingredient compound was downloaded from PubChem database (https://pubchem.ncbi.nlm.nih.gov/docs/about/, accessed on 18 December 2024) and the three-dimensional crystal structure of the target protein (SUT) was downloaded from the PDB database (https://www.rcsb.org/, accessed on 28 December 2024). Sitemap was used for active site prediction. It was processed using Schrodinger’s Protein Preparation module, including residue repair, hydrogen bond optimization, solvent removal, and energy minimization. The ligand was prepared using the LigPrep module with OPLS3e force field and ionized and minimized. The preprocessed protein and ligand were then docked using the Ligand Docking module, selecting the prepared Grid and Lig file, and using the Glide module for standard precision docking. After clicking “Run”, the docking results were obtained. Finally, the results were visualized using Pymol.

The structures of active ingredient compounds were downloaded from PubChem database and imported into ChemBio3D 14.0 software to adjust the spatial conformation of active ingredients and calculate the optimization of energy. After AutoDockTools 1.5.6 processing, the target protein was downloaded from the Uniprot database and imported into AutoDockTools1.5.6 for hydrogenation, charge distribution, and atomic type addition [45]. AutoDockVina was used for molecular docking, and the docking results were plotted with Pymol [46,47]. Molecular docking was performed using the DOCK 6.9 software suite developed by the University of California, San Francisco (UCSF) to predict the binding orientation and affinity between ligands and the target protein. The crystal or AlphaFold-predicted structure of the receptor was prepared by removing all heteroatoms and water molecules, adding polar hydrogens, and assigning AMBER united atom charges using UCSF Chimera (Resource for Biocomputing, Visualization, and Informatics, University of California, San Francisco, CA, USA) [48,49,50].

### 4.3. Phylogenetic Relationships, Conserved Motifs, and Gene Structures of the DrSUT Gene Family

To investigate the phylogenetic relationships of *SUT* genes, SUT protein sequences from *Arabidopsis thaliana* and *Dioscorea rotundata* were retrieved from the UniProt database (https://www.uniprot.org) for the construction of a neighbor-joining (NJ) phylogenetic tree. The SUT protein sequences from all plant species were aligned using ClustalX 1.81. The phylogenetic tree, including multiple plant species (*Arabidopsis thaliana and Dioscorea rotundata*), was constructed using Mega11.0 with the NJ method, selecting the Poisson model and performing 1000 bootstrap replications for validation. Prior to tree construction, we aligned the amino acid sequences of *DrSUT* and *AtSUT* genes using ClustalX 1.81.

The DNA and cDNA sequences of the *DrSUT* gene were used to predict intron structure via the online Gene Structure Display Server (GSDS) 2.0 (https://gsds.gao-lab.org/Gsds_help.php, accessed on 18 December 2024). Conserved motifs of SUT proteins, including all *DrSUT* sequences, were identified using the MEME Suite (https://meme-suite.org/meme/, accessed on 18 December 2024) [51]. The parameters were set as follows: the motif width was set between 6 and 50 amino acids; the maximum number of motifs was set to 20; and the number of motif occurrences per sequence was set to “any”, with no limitations. The resulting motifs were visualized using TBtools software (v2.310). The genome sequence and annotation files of *Dioscorea rotundata* were downloaded from the NCBI database. The gene structures of *DrSUT* family members were illustrated using the “Gene Structure View” module in TBtools.

### 4.4. Chromosomal Mapping, Gene Replication, and Syntenic Analysis with Other Plant Species

The physical locations of *DrSUTs* were obtained in the genome annotation file downloaded from the Yam Genomics database and visualized with TBtools. The collinear relationships of *DrSUT* genes were analyzed with Dual Synteny Plotter software(MCScanX v1.0.0, accessed on 25 March 2025). The tandem replication and segmental replication events in the *DrSUT* genes were analyzed by multiple collinear scanning toolkits (MCScanX v1.0.0), *DrSUT* genes were found to be located on the 8 chromosomes of *Dioscorea rotundata*. The SUT collinearity pairs between *Dioscorea rotundata* and five other species—*Dioscorea alata*, *Dioscorea zingiberensis*, sweet potato (*Ipomoea batatas*), rice (*Oryza sativa*), and *Arabidopsis thaliana*—were extracted using TBtools and used to construct collinearity maps [52].

### 4.5. Identification of Cis-Regulatory Elements in the Promoter Regions of SUT Genes

TBtools was used to extract the DNA sequence 2000 bp upstream of the *SUT* promoter region in the yam’s genome. These genes were submitted to the PlantCARE database (http://bioinformatics.psb.ugent.be/webtools/plantcare/html/, accessed on 28 April 2024) [53], cis-acting elements were identified, and stress response, plant growth and development, and hormone response elements were screened. GO annotation analysis was conducted by extracting the DNA sequences 2000 bp upstream of the psmyb coding sequences using the GXF Sequences Extract tool in TBtools. Eggnog (http://eggnog5.embl.de/, accessed on 28 April 2024) [54] was used for the GO annotation analysis, and the results were visualized using WeGo (https://wego.genomics.cn/, accessed on 28 April 2024) [55].

### 4.6. Expression of the DrSUT Genes According to Quantitative Real-Time Polymerase Chain Reaction (qRT-PCR)

Transcriptome analysis was performed utilizing publicly accessible databases. RNA-seq datasets for yam (*Dioscorea rotundata*), representing various tissue types—including young stem, mature stem, middle tuber, and tuber apex—were obtained from the NCBI Sequence Read Archive under project ID PRJDB3383 (https://www.ncbi.nlm.nih.gov/sra/?term=DRR063126, accessed on 18 December 2024). This analysis aimed to explore gene expression profiles across different yam tissues. Heatmaps illustrating differential gene expression were generated using the OmicStudio platform (https://www.omicstudio.cn/home, accessed on 28 April 2024). Additionally, the transcription levels in the fruits at seven developmental stages (90, 105, 120, 135, 150, 165, and 180 days after DAP) were also assessed. The qRT-PCR primers for the *DrSUT* genes were designed using Primer Premier 5.0 (PREMIER Biosoft International, Palo Alto, CA, USA) and the specific primer information is provided in Table S4. The primers used in our experiments were synthesized by Sangon Biotech (Shanghai) Co., Ltd. (Shanghai, China) The company’s website is: https://www.sangon.com. qRT-PCR analysis was conducted using SYBR^®^ Premix Ex Taq™ II (Tli RNaseH Plus, RR820A; TaKaRa Biotechnology, Dalian, China) on an FTC-3000P system (Funglyn Biotech, Toronto, ON, Canada), according to the manufacturer’s instructions. Gene expression levels were calculated using the 2^−ΔΔCT^ method, with UBQ as the internal control. The experiments were conducted with three biological replicates, and three technical replicates were performed for each biological replicate [56]. Moreover, total RNA was treated with DNase I (TaKaRa,Biotechnology, Dalian, China) prior to reverse transcription to eliminate potential genomic DNA contamination. Sugar content in tubers at different developmental stages was determined with reference to the previously described method [57].

### 4.7. DrSUT1 Transport Activity and Expression in Heterologous Systems

The recombinant plasmid *PDR-DrSUT1* was transformed into the EBY.VW4000 yeast strain. The transformed recombinant yeast cells were inoculated into liquid YPA medium containing 2% (*w*/*v*) maltose and cultured at 30 °C with shaking at 220 rpm until the cell density reached an OD_623_ of 0.8 [58], then collected by centrifugation (2400× *g*) and washed twice with 25 mM phosphate-buffered saline (PBS, pH 5.5). Cells were resuspended in PBS to an OD_623_ of 20 for uptake analysis. [^14^C]-sucrose (0.02 μCi) was added to the suspension, yielding a final sucrose concentration of 100 mM. After incubation at 30 °C with shaking for the indicated time, cells were washed three times with 1 mL cold distilled water. Radioactivity was measured by adding 0.5 mL scintillation fluid-Eccoscint H,Thermo Fisher Scientific (Waltham,MA,USA)and counting with a liquid scintillation counter (Tri-Carb 2810 TR, PerkinElmer, Waltham, MA, USA).Seeds of *Arabidopsis thaliana*, including *DrSUT1* transgenic lines and non-transgenic controls, were surface-sterilized by soaking in 70% ethanol for 10 min, followed by treatment with a sodium hypochlorite solution for 20 min. After sterilization, the seeds were plated on 1/2 MS solid medium. The seeds were stored at 4 °C for 3 days to break dormancy and then transferred to a growth chamber maintained at 24 °C under a 16-h light/8-h dark photoperiod with 70% relative humidity. Once the seedlings had developed approximately four true leaves, they were transplanted into soil. The seedlings were grown vertically on 1/2 MS solid medium in 10 cm × 10 cm square plates. Root length was measured using a vernier caliper after 14 days.

## 5. Conclusions

This study provides a genome-wide characterization of the *DrSUT* gene family in *Dioscorea rotundata*, identifying eight members with conserved gene structures, motifs, and functional domains. Phylogenetic and evolutionary analyses revealed that most *SUT* genes are under strong purifying selection, with limited functional divergence observed in certain members, such as *DrSUT6*. Expression profiling across different tissues and developmental stages demonstrated that *DrSUT1*, *DrSUT3*, and *DrSUT8* are highly expressed during tuber initiation and expansion, indicating their potential roles in sucrose unloading and accumulation. Molecular docking analyses revealed conserved residues involved in hydrogen bonding with sucrose, supporting their function in high-affinity transport. Promoter analysis identified various cis-regulatory elements responsive to hormones, abiotic stress, and light, suggesting dynamic transcriptional regulation. Additionally, GO enrichment and complementary expression patterns with *SWEET* genes point to a coordinated network in regulating sucrose partitioning and long-distance transport. Together, these findings enhance our understanding of sucrose transport mechanisms in yam and provide valuable genetic resources for future molecular breeding aimed at improving tuber yield and carbohydrate allocation efficiency in *Dioscorea rotundata*.

## Figures and Tables

**Table 1:** The information of the *DrSUT* gene family.

Gene Name	Sequence ID	Number of Amino Acid	Molecular Weight	Theoretical pI	Instability Index	Aliphatic Index	Grand Average of Hydropathicity	Subcellular Localization
*DrSUT1*	XP_039125404.1	616	66,142.36	6.42	37.84	94.43	0.294	Plasma Membrane
*DrSUT2*	XP_039125505.1	504	54,428.15	8.62	40.47	109.52	0.638	Plasma Membrane
*DrSUT3*	XP_039137042.1	501	53,651.15	9.19	38.62	114.13	0.596	Plasma Membrane
*DrSUT4*	XP_039137043.1	501	53,651.15	9.19	38.62	114.13	0.596	Plasma Membrane
*DrSUT5*	XP_039144966.1	511	55,381.61	8.78	32.7	99.86	0.515	Plasma Membrane
*DrSUT6*	XP_039144965.1	507	54,596.84	8.58	30.15	103.51	0.565	Plasma Membrane
*DrSUT7*	XP_039145101.1	513	55,240.9	8.78	33.65	103.47	0.551	Plasma Membrane
*DrSUT8*	XP_039145776.1	510	55,188.44	8.89	36.74	102.14	0.559	Plasma Membrane

## Data Availability

Data will be made available on request.

## Supplementary Materials

The supporting information can be downloaded at: https://www.mdpi.com/article/10.3390/ijms26125756/s1.
