# Supplementary material for: Genome-Wide Identification, Plasma Membrane Localization, and Functional Validation of the SUT Gene Family in Yam (Dioscorea cayennensis subsp. rotundata)
Source: Int J Mol Sci. 2025 Jun 16;26(12):5756. doi: 10.3390/ijms26125756 (PMC12193089; doi:10.3390/ijms26125756)

Docking results
sut

Results of maestro

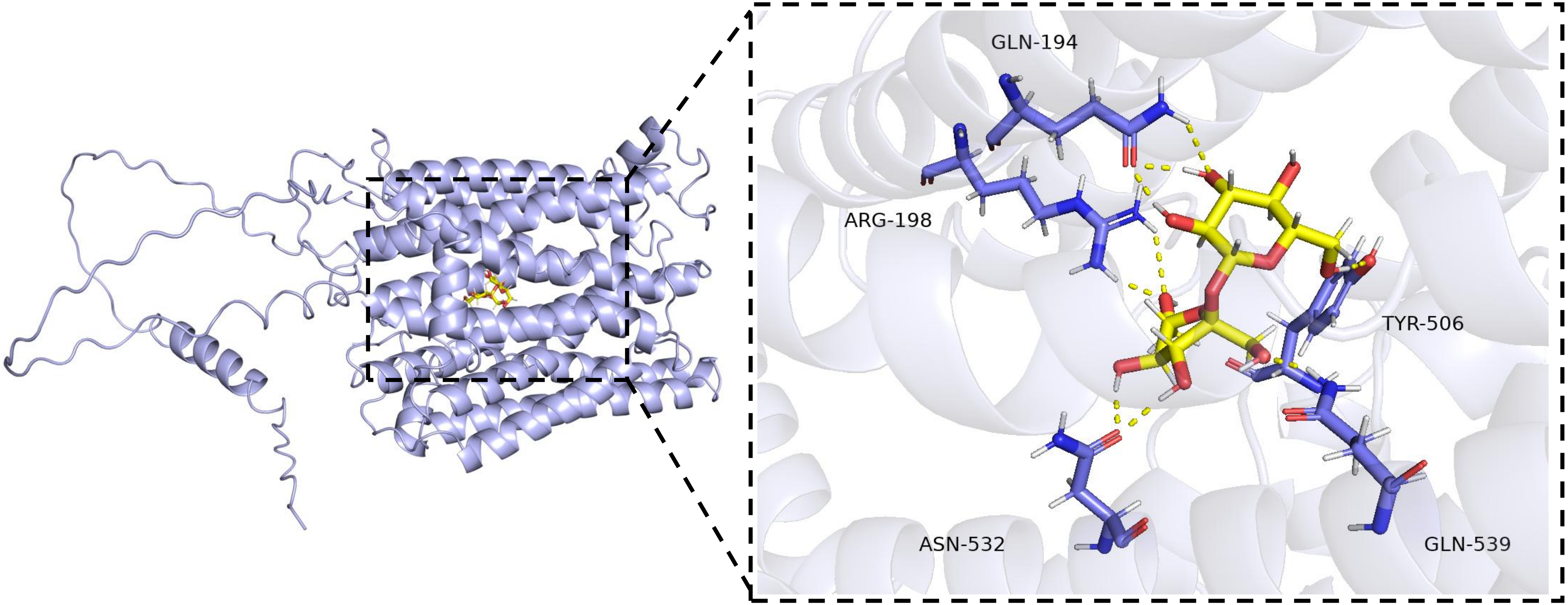

3D Binding model analysis of SUT protein (blue) with compound Sucrose(yellow).
The key residue are shown sticks.
H-bonds are shown as yellow dashed line.
Binding energy: -7.868 kcal/mol

Results of Dock

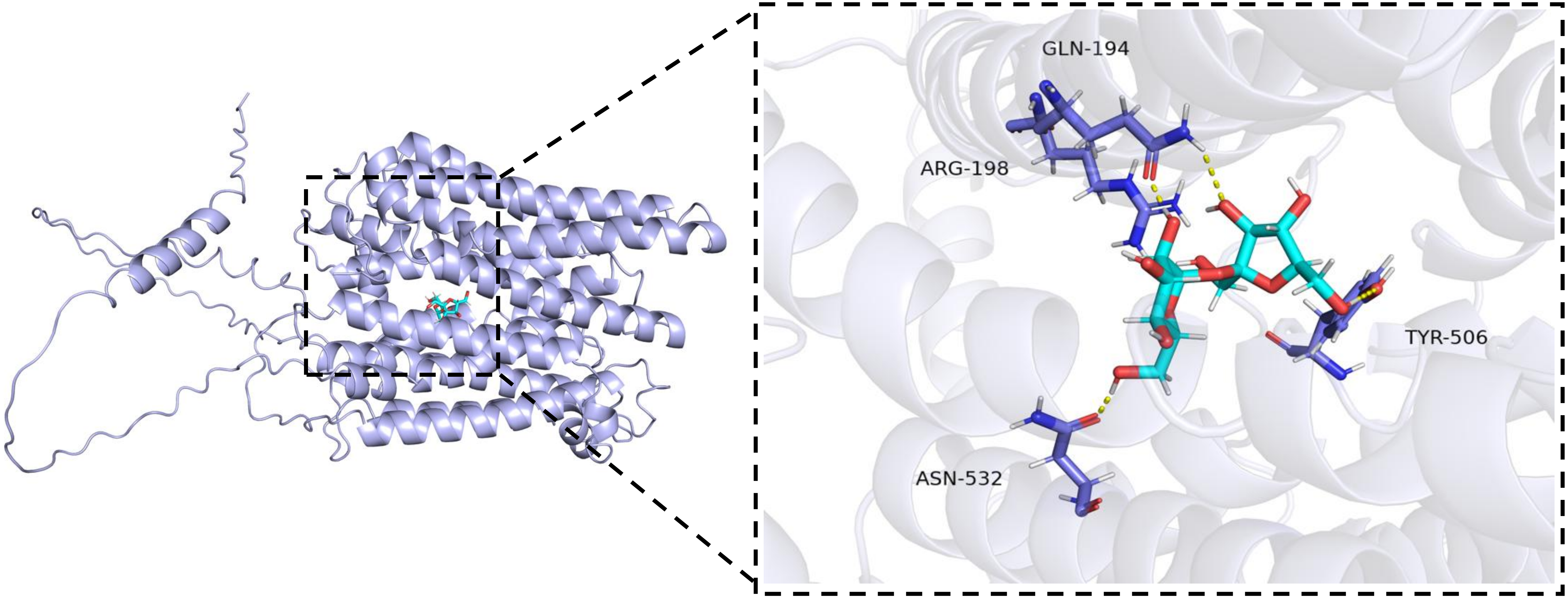

3D Binding model analysis of SUT protein (blue) with compound Sucrose (cyan).
The key residue are shown sticks.
H-bonds are shown as yellow dashed line.
Binding energy: -6.158 kcal/mol

Results of Autodock Vina

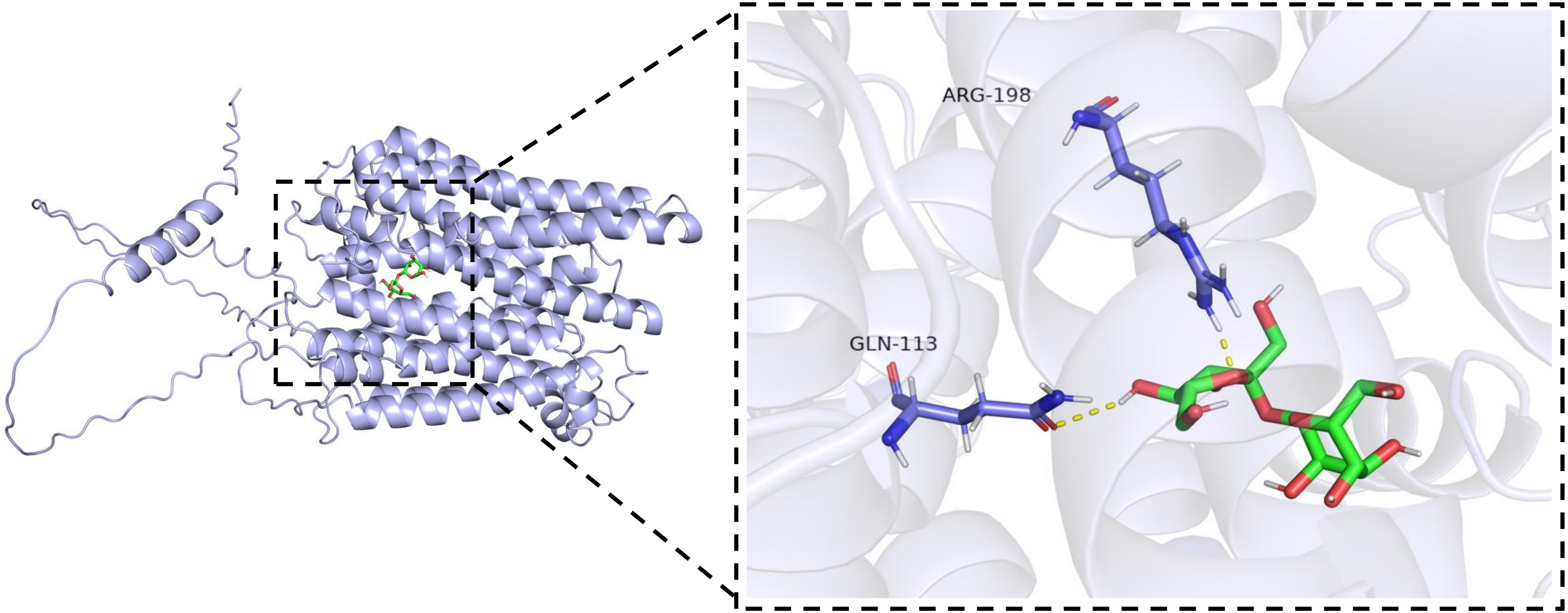

3D Binding model analysis of SUT protein (blue) with compound Sucrose (green).
The key residue are shown sticks.
H-bonds are shown as yellow dashed line.
Binding energy: -6.411 kcal/mol

The active pockets of the protein were identified using SiteMap, and the results are shown in the table below.

Pocket ID	Site score	Size	Volume
1	1.121	434	1338.043
2	1.081	234	378.672
3	0.814	43	88.837
4	0.661	28	154.693

The binding modes from the three docking software programs are shown in the figure above: yellow (Maestro), cyan (DOCK), and green (AutoDock Vina), with the red region indicating the active site. As shown in the figure, the binding regions of the three modes are closely aligned. Furthermore, all three docking poses are located within the highest-confidence active pocket (Pocket 1).

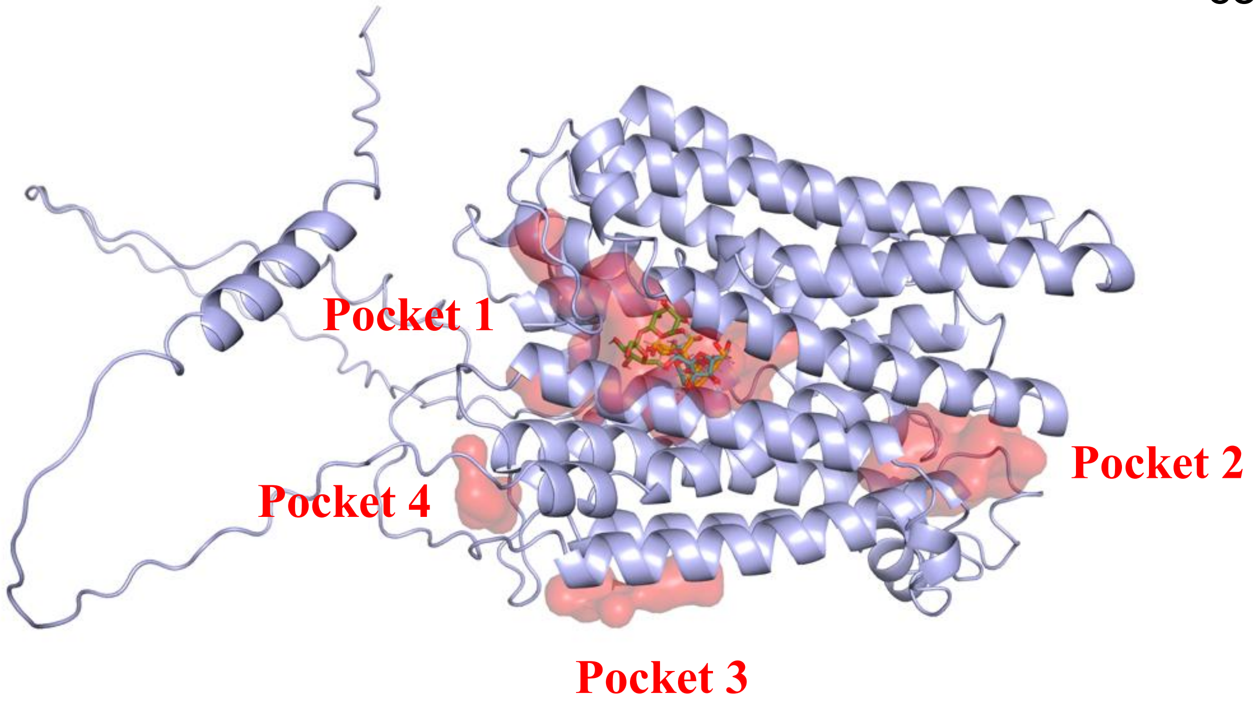

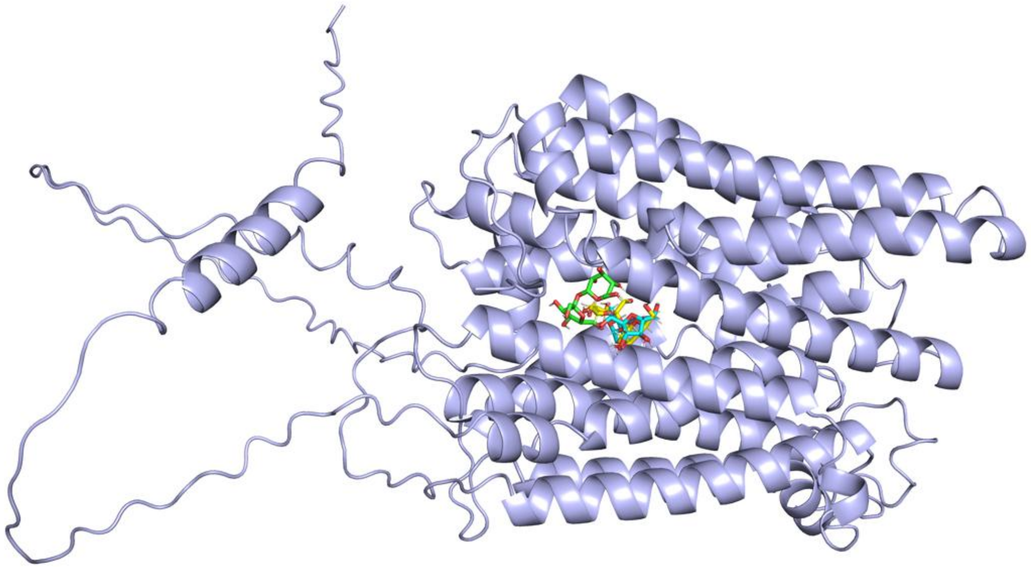

Supplement: Supplementary file 1 [file ijms-26-05756-s001.zip › Figure S1.pdf]
