# Supplementary figures and images for: Genome-Wide Identification, Plasma Membrane Localization, and Functional Validation of the SUT Gene Family in Yam (Dioscorea cayennensis subsp. rotundata)
Source: Int J Mol Sci. 2025 Jun 16;26(12):5756. doi: 10.3390/ijms26125756 (PMC12193089; doi:10.3390/ijms26125756)

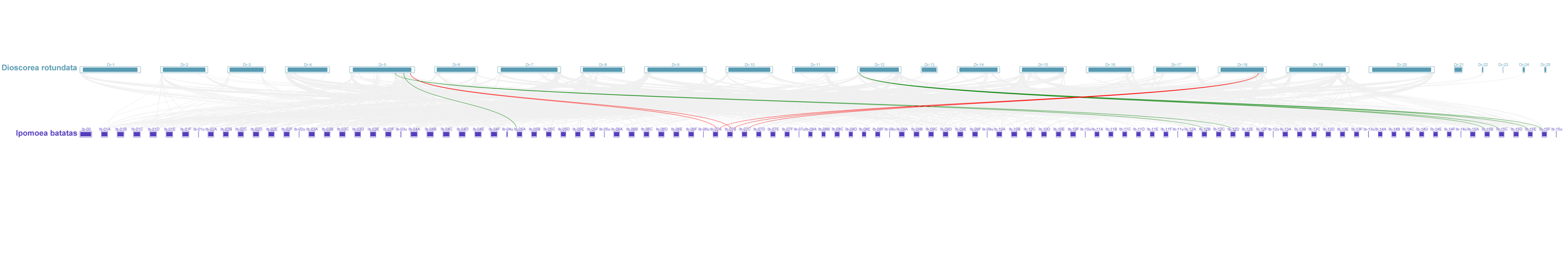

Supplement: Supplementary file 1 [file ijms-26-05756-s001.zip › Figure S2.tiff]
